# Barriers to patient recruitment in a poststroke neurorehabilitation multicenter trial in Brazil

**DOI:** 10.1590/1414-431X2023e12326

**Published:** 2023-01-27

**Authors:** T.R. da Silva, G.J. Luvizutto, L.G. Martins, R.D.M. da Costa, J.T. de Souza, F.C. Winckler, L.C.A. Sartor, G.P. Modolo, N.C. Ferreira, J.C.S. Rodrigues, R.G. Kanda, M.O. Fogaroli, G.F. Borges, G.R.S. Rizzatti, P.W. Ribeiro, D.S. Pires, D.B. Favoretto, L.R. Aguiar, S.G.Z. Bazan, L.E.G. Betting, L.C.O. Antunes, H.R.C. Nunes, V.M. Pereira, T.G.S. Edwards, O. Pontes-Neto, A.B. Conforto, R. Bazan

**Affiliations:** 1 Departamento de Clínica Médica, Faculdade de Medicina de Botucatu, Universidade Estadual Paulista, Botucatu, SP, Brasil; 2 Departamento de Fisioterapia Aplicada, Universidade Federal do Triângulo Mineiro, Uberaba, MG, Brasil; 3 Departamento de Reabilitação, Hospital das Clínicas, Faculdade de Medicina de Botucatu, Universidade Estadual Paulista, Botucatu, SP, Brasil; 4 Departamento de Neurologia, Psicologia e Psiquiatria, Faculdade de Medicina de Botucatu, Universidade Estadual Paulista, Botucatu, SP, Brasil; 5 Hospital das Clínicas, Universidade de São Paulo, São Paulo, SP, Brasil; 6 Departamento de Neurociências e Ciências do Comportamento, Hospital das Clínicas, Faculdade de Medicina de Ribeirão Preto, Universidade de São Paulo, Ribeirão Preto, SP, Brasil; 7 Departamento de Saúde Pública, Faculdade de Medicina de Botucatu, Universidade Estadual Paulista, Botucatu, SP, Brasil; 8 Department of Neurology, University of Toronto, Toronto, Ontario, Canada

**Keywords:** Stroke, Unilateral spatial neglect, Transcranial direct current stimulation, Rehabilitation, Clinical trial

## Abstract

There is a high demand for stroke rehabilitation in the Brazilian public health system, but most studies that have addressed rehabilitation for unilateral spatial neglect (USN) after stroke have been performed in high-income countries. Therefore, the aim of this study was to analyze USN patient recruitment in a multicenter noninvasive brain stimulation clinical trial performed in Brazil and to provide study design recommendations for future studies. We evaluated the reasons for exclusion of patients from a multicenter, randomized, double-blinded clinical trial of rehabilitation of USN patients after stroke. Clinical and demographic variables were compared between the included and excluded patients. A descriptive statistical analysis was performed. Only 173 of the 1953 potential neglect patients (8.8%) passed the initial screening. After screening evaluation, 87/173 patients (50.3%) were excluded for clinical reasons. Cognitive impairment led to the exclusion of 21/87 patients (24.1%). Low socioeconomic status led to the exclusion of 37/173 patients (21.4%). Difficulty obtaining transportation to access treatment was the most common reason for their exclusion (16/37 patients, 43.3%). The analyzed Brazilian institutions have potential for conducting studies of USN. The recruitment of stroke survivors with USN was restricted by the study design and limited financial support. A history of cognitive impairment, intracranial stenting or craniectomy, and lack of transportation were the most common barriers to participating in a multicenter noninvasive brain stimulation trial among patients with USN after stroke.

## Introduction

Stroke is the second leading cause of death and disability worldwide, mainly occurring in low- and middle-income countries (1,2). After a stroke, patients can develop sensory impairments that affect their ability to direct their attention to visual, auditory, or tactile stimuli. Since different types of neglect can occur, several terms are used in clinical practice, such as unilateral spatial neglect (USN), motor neglect, hemineglect, and inattention (3).

USN designates a consistent, exaggerated spatial asymmetry in processing information in peri-personal and/or extra-personal space due to an acquired cerebral lesion, more frequently associated with right hemisphere strokes (4-6). Approximately 43% of poststroke patients with right hemisphere lesions present with USN (7-10), leading to negative impacts on their functional capacity, social participation, and quality of life (11-13).

Many individuals with USN after stroke have major functional disabilities as well as decreased rates of adherence to rehabilitation programs and independence in self-care skills (14,15). In light of these functional implications, it is not surprising that rehabilitation using noninvasive brain stimulation for USN is an important topic in stroke rehabilitation research. However, until now, trials have been limited by small samples and insufficient methodological quality and performed in high-income countries with better infrastructure and funding (16-18). Geographic location and sociodemographic factors play important roles in access to health care and may be crucial in the successful treatment from acute care to poststroke rehabilitation (19). The results of these trials are often not applicable to low- and middle-income countries due to economic, social, and cultural differences.

The development of rehabilitation strategies that are more cost-effective and accessible to the entire population has been a major objective of research related to the rehabilitation of poststroke patients in low- and middle-income countries. Based on this premise, it is essential to conduct clinical studies with feasible treatment strategies in developing countries so that evidence-based services can be structured taking into account the personal, social, and demographic characteristics of the population. Clinical study protocols should be developed to identify and understand the difficulties in recruitment and the reasons for participant exclusion in low- and middle-income countries. Therefore, this study aimed to identify the characteristics and main barriers to patient participation in a research protocol performed in a developing country.

## Material and Methods

### Context

We prospectively evaluated the reasons for exclusion of patients from a multicenter, randomized, double-blinded clinical trial performed at the Botucatu Medical School, Ribeirao Preto Medical School, University of São Paulo, and Toronto Western Hospital. The trial protocol was approved by the National Regulatory Agency in Research and the Institutional Review Board of each participating site: Botucatu Medical School (CAAE: 41171315.8.0000.5411); Ribeirao Preto Medical School (CAAE: 41171315.8.2001.5440); University of São Paulo (CAAE: 41171315.8.2002.00680); Toronto Western Hospital (CAAE: 41171315.8.1001.5411). The trial was registered in the Brazilian Registry of Clinical Trials (RBR-78jvzx) on April 20th, 2020.

The data of this study originated from the ELETRON trial, a pilot double-blinded, multicenter randomized clinical trial that enrolled patients with USN after ischemic stroke (20). The trial was performed in three hospitals in different cities in southern São Paulo state. The main center for the trial (Botucatu Medical School - Center 1) has 490 beds, including a 10-bed stroke unit. The Clinics Hospital of the University of São Paulo (Center 2), the largest hospital in São Paulo, has 2200 beds and a stroke unit with 10 beds. The Ribeirao Preto Medical School (Center 3) has 922 hospital beds and a stroke unit with 10 beds. A researcher from the Toronto Western Hospital (Center 4) was responsible for monitoring adverse events in the trial. All stroke units had a mixed and comprehensive model involving diagnostic and intervention strategies from the acute phase until rehabilitation. Each center managed their own research activities.

### Recruitment

Patients from Centers 1, 2, and 3 were enrolled and randomly assigned to one of three groups, i.e., anodal transcranial direct current stimulation (A-tDCS), cathodal transcranial direct current stimulation (C-tDCS), and sham transcranial direct current stimulation (S-tDCS). Randomization was performed by a research assistant using a real-time, dynamic, internet-based, randomized minimization procedure to balance the numbers of patients across the three groups with respect to age, baseline National Institutes of Health Stroke Scale (NIHSS) score, use or non-use of intravenous alteplase, baseline Behavioral Inattention Test-conventional (BIT-C) score, and participating centers. A second research assistant opened consecutively numbered, randomly ordered, opaque envelopes containing the group allocation results after the baseline assessment. Assessments of outcomes were performed by an assessor blinded to the treatment allocation results, who conducted a detailed assessment of the participants’ conditions for the training program.

### Interventions

In this trial, patients with USN after stroke were randomized into three groups of treatment: A-tDCS of the right somatosensory cortex (P4), C-tDCS of the left somatosensory cortex (P3), or S-tDCS associated with 15 sessions of physical therapy, 2 times per week, for 7.5 weeks. A direct current was delivered by a battery-powered device (DC-Stimulator Plus model, NeuroConn, neuroCare Group GmbH, Germany) using two pairs of surface saline-soaked sponge electrodes (5×5 cm) (21). For real stimulation, a constant current of 1 mA was delivered for 20 min. For the sham condition, the stimulator was turned on and the current intensity was gradually increased for 30 s and then tapered off over 30 s.

### Study of the interventions

Patients admitted to the neurology emergency room, stroke unit, or neurovascular clinic were screened for inclusion from February 2017 to June 2020 by evaluation of their medical files and interviews about their clinical conditions. The research staff had direct access to clinical charts to screen patients for potential eligibility. The inclusion and exclusion criteria are described in Table 1.

**Table 1:** ELETRON trial inclusion and exclusion criteria.

Inclusion criteria	Exclusion criteria
• Stroke diagnosis at least 3 weeks and at most 6 months, with ischemia or hemorrhage in the right hemisphere confirmed by computed tomography or magnetic resonance imaging	• Presence of metal in the cranial cavity (stent, clip)• Lesions in the electrode placement area• Previous surgery on the skull, eyes or craniectomy
• Presence of USN confirmed by BIT-C*	• Epileptic crisis
• Both sexes	• Clinical instability
• Age over 18 years	• Severe cognitive impairment
	• Global aphasia
	• Visual impairments
	• Other associated neurological diseases
	• Possibility of pregnancy
	• Amputation of lower limbs or upper limbs
	• Disabling pain
	• Lack of interest in participating in the study
	• Difficulty with transportation to access the treatment site
	• Social or economic difficulty
	• Difficulty in establishing telephone contact with patient or caregiver

USN: unilateral spatial neglect; BIT-C: Behavioral Inattention Test-conventional. *Cut-off score for the BIT-C is <129 for confirming diagnosis of USN.

If the inclusion criteria were fulfilled, patients were contacted by phone after hospital discharge to schedule an evaluation to confirm their eligibility. After inclusion, three evaluations were performed, one at the beginning, one in the middle (8 sessions), and another at the end of the protocol (15 sessions). The patient had to attend the rehabilitation center 16 times during the trial. Patients were dropped from the study if there were significant adverse events, death, clinical instability [systolic blood pressure >220 mmHg, oxygen saturation <92%, resting heart rate <40 or >110 beats per min, temperature >38.5°C, hemoglobin <7 g/dL, platelets <50 × 10^9^/L (22)], three consecutive absences, or nonadaptation to the protocol.

### Measures

Clinical and demographic variables were evaluated in all included and excluded patients. Age was evaluated by interviewing the patient or the legal guardian. Race, medical history, and hospital information were determined based on patient reports. The main outcome of this study was the reason for patient exclusion from the clinical trial. This outcome was divided by clinical (any medical information that excluded patients according to the trial protocol), technical (tDCS equipment failure), or socioeconomic reasons (difficulty with transportation, lack of caregivers to accompany them to sessions, lack of telephone contact, low interest in participating in the research, or failure to attend the scheduled evaluations).

### Sample size

A sample size of 15 patients per group was estimated assuming type I and II error probabilities of 0.05 and 0.20, respectively, normal distribution for the outcomes, reduction of 15% in the USN degree evaluated by the line cancellation test after A-tDCS, and a variation coefficient of 20% based on implemented computer simulations.

### Analysis

Descriptive statistical analysis was performed. Continuous data are reported as the median and interval (minimum and maximum). Categorical data are reported as frequencies. Clinical and demographic variables were compared between included and excluded patients using the Mann-Whitney test for continuous variables and the chi-squared test for categorical variables. All statistical analyses were performed using IBM SPSS Statistics for Windows/Macintosh, version 24.0 (USA).

### Ethical considerations

This study was approved by an ethics committee and/or followed the tenants of the Declaration of Helsinki. The enrolled patients or their guardian provided written informed consent.

## Results

**Figure 1:**
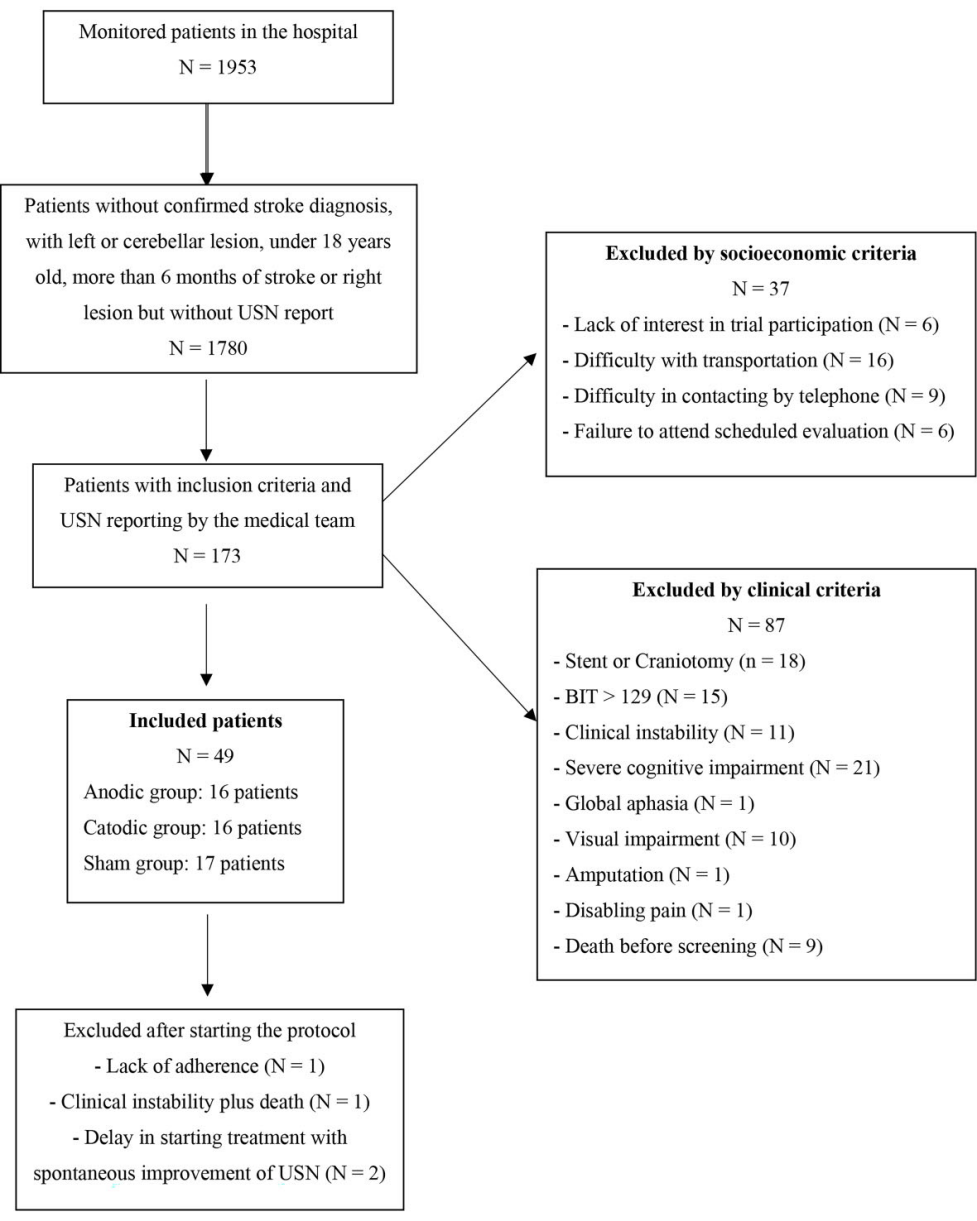
ELETRON Trial's flowchart. USN: unilateral spatial neglect; BIT: Behavioral Inattention Test.

The trial flowchart is shown in Figure 1. A total of 1953 subjects were recruited for our study. A total of 1780 did not meet the inclusion criteria, and 173 (8.8%) passed the initial screening. A total of 124 (69.3%) patients were excluded, 49 were included in the protocol, and 45 completed the trial. The clinical and demographic characteristics of the included and excluded patients are shown in Table 2.

**Table 2:** Demographic and clinical history of included and excluded patients.

Variables	Included patients (n=49)	Excluded patients (n=124)	P
Demographic variables			
Age (years)	64.5 (35.0-82.0)	68.0 (52.0-86.0)	0.495
Male sex, n (%)	27 (55.1)	72 (58.1)	0.736
White race, n (%)	38 (77.5)	98 (79.0)	0.839
Medical history (%)			
History of ischemic stroke/TIA, n (%)	4 (8.2)	24 (19.3)	0.107
Diabetes mellitus, n (%)	28 (57.1)	70 (56.4)	>0.99
Hypertension, n (%)	40 (81.6)	94 (75.8)	0.545
Current or past tobacco use, n (%)	30 (61.2)	66 (53.2)	0.495
Obesity, n (%)	15 (30.6)	43 (34.7)	0.596
Heart disease, n (%)	3 (7.5)	22 (17.7)	0.056
Hospitalization			
Thrombolysis, n (%)	8 (16.3)	19 (15.3)	>0.99
Time of hospitalization (days)	9.5 (0.0-15.0)	14.0 (1.0-30.0)	0.127
NIHSS (hospital discharge)	4.0 (0.0-15.0)	8.0 (6.0-18.0)	0.270
mRS (hospital discharge)	3.0 (1.0-5.0)	4.0 (2.0-5.0)	0.239

Data are reported as median and intervals, unless otherwise indicated. Mann-Whitney test or chi-squared test. TIA: transient ischemic attack; NIHSS: National Institutes of Health Stroke Scale (for stroke severity, score range from 0 to 42, with higher values indicating worse stroke severity); mRS: modified Rankin scale (for functional incapacity, score range from 0 to 6, with higher values indicating worse functional capacity).

The recruitment for the study by Centers 1, 2, and 3 is shown in Table 3. Reasons for exclusion in each center are shown in Table 4.

**Table 3:** Recruitment carried out by Centers 1, 2, and 3.

Center	RP	S	IC	EC	IP	EP
1	48 months	1033	143	97	47	5
2	12 months	336	16	15	0	0
3	12 months	584	14	12	2	0

RP: recruitment period; S: patients screened; IC: patients with inclusion criteria; EC: patients with exclusion criteria; IP: included patients; EP: excluded patients after protocol inclusion.

**Table 4:** Reasons for exclusion of patients recruited at Centers 1, 2, and 3.

Exclusion criteria	Center 1	Center 2	Center 3	Total	%
Clinical criteria (n = 87)					
Severe cognitive impairment, decreased level of consciousness	17	0	4	21	24.1
Stent or craniectomy	18	0	0	18	20.7
BIT >129 (spontaneous improvement of USN)	15	0	0	15	17.2
Clinical instability	7	0	4	11	12.6
Visual impairment	9	1	0	10	11.5
Death before screening	9	0	0	9	10.3
Disabling pain	0	0	1	1	1.1
Amputation	1	0	0	1	1.1
Aphasia	1	0	0	1	1.1
Socioeconomic criteria (n = 37)					
Difficulty with transportation	8	5	3	16	43.3
Difficulty in phone contact	5	3	1	9	24.3
Lack of interest	2	1	3	6	16.2
Failure to attend scheduled evaluation	4	2	0	6	16.2

BIT: Behavioral Inattention Test.

After screening, 87 of 173 patients (50.3%) were excluded for clinical reasons, and 21.4% (37 patients) were excluded for socioeconomic reasons. The main clinical reasons for exclusion were cognitive impairments (n=21/87, 24.1%), presence of intracranial stent or craniectomy surgery (n=18/87, 20.7%), spontaneous improvement of USN between hospital discharge and screening (n=15/87, 17.2%), and clinical instability (n=11/87, 12.6%). Other less frequent factors were global aphasia, visual disturbances, amputations, and deaths after hospital discharge.

Among the socioeconomic factors, the most common reasons were difficulty with transportation to the hospital (n=16/37; 43.3%) and difficulty establishing phone contact with the patient and caregiver (n=9/37; 24.3%). In addition, there was a lack of interest in participating in the study (n=6/37; 16.2%) and failure to attend scheduled evaluations (n=6/37; 16.2%).

After inclusion in the protocol, four patients were excluded (1 due to difficulty in adhering to treatment, 1 due to clinical instability, and 2 due to spontaneous improvement of USN).

## Discussion

In this study, we observed that the main barriers to participation in a randomized clinical trial of noninvasive brain stimulation to treat USN in a developing country were clinical barriers, such as spontaneous USN recovery between the hospital discharge and start of the trial, clinical instability, significant cognitive changes, and limitations for tDCS applications. In addition, social barriers were identified, as a considerable proportion of patients had socioeconomic problems related to difficulty with transportation, lack of caregivers to accompany them to sessions, lack of telephone contact, low interest in participating in the research, or failure to attend scheduled evaluations.

Difficulties in identifying the neurological recovery phase and standardization of terminology are frequently reported in clinical trials of rehabilitation in stroke patients (23). This trial had relatively comprehensive inclusion criteria, and only 8.8% of the screened subjects were included. The low prevalence of USN should be considered (7), given that it is a condition that is underdiagnosed (24) and causes high dependency and poor functionality (11-13). These USN characteristics lead to difficulties for the patient in accessing the rehabilitation center. However, several patients had spontaneous improvement of USN before trial screening. Farnà et al. (25) observed that spatial attention deficits partially improved during the acute phase of stroke in less than half of USN patients. On the other hand, clinical instability and significant cognitive changes were frequently observed in these patients before trial enrollment. USNs are frequently associated with structural damage in a dorsal frontoparietal network that controls spatial attention with important limitations of cognitive function (26). These sequelae limited the scale application and ability to participate in the trial.

Ko et al. (27), in a double-blind, crossover, sham-controlled experiment, evaluated the effects of anodal tDCS over the right parietal cortex on USN patients after stroke. Subjects who had metal in the cranial cavity or calvarium or skin lesions in the area of the electrode and patients who had uncontrolled medical problems and severe cognitive impairments were excluded. Sunwoo et al. (28) evaluated the effects of dual and single-mode tDCS over the bilateral parietal cortex on USN in stroke patients in a clinical trial. The authors excluded patients who had metallic implants in the cranial cavity, a skull defect, a history of seizure, uncontrolled medical problems, and severe cognitive impairment. In both studies (27,28), the exclusion criteria were the same as in the ELETRON trial, but they did not report the number of excluded patients. An additional barrier in this trial was factors that interfered with the safe application of tDCS: the presence of a metallic implant in the cephalic region, decompressive craniectomy, and injury in the electrode placement area. USN is frequently observed in patients with severe stroke lesions that commonly progresses to decompressive craniectomy and clinical instability.

Despite not being formal exclusion criteria, our study also demonstrated important socioeconomic barriers, such as difficulty with transportation and the absence of caregivers to accompany the patients to tDCS and physical therapy sessions. In the original protocol proposal (21), the trial was to be conducted at a single center. However, with the difficulties of patient recruitment, three other stroke centers aided in screening, selection, treatments, and safety monitoring (22). Other studies have also shown that there are barriers to rehabilitation trials in developing countries, such as stroke recurrence, chronic uncontrolled diseases, social difficulties, and access to rehabilitation services (29). Scianni et al. (30) also observed that lack of transport was the most common barrier to participation in training sessions of a rehabilitation stroke clinical trial in Brazil. Bernhardt et al. (23) reported that the recruitment in developing countries is more difficult due to the socioeconomic impact compared to developed countries.

In addition, the failure to attend scheduled evaluations and lack of interest in trial participation and adherence to treatment negatively impacted the study protocol. Health in developing countries is not considered a priority, as the patient and caregiver often do not recognize the importance of rehabilitation due to the lack of an adequate support network for health information (31). Considering the local characteristics of different centers, patients from larger cities, such as Ribeirão Preto and São Paulo, have difficulty to access the rehabilitation center, which makes it difficult to comply with the protocol schedule. The difficulty of access may be due to architectural barriers in the city, transport difficulties, and lack of financial support. In this trial, none of the centers provided transportation, which could decrease adherence rates during treatment.

We observed that both clinical and socioeconomic factors acting as barriers to rehabilitation trial participation in developing countries are modifiable by public health policies, constant training of health teams, and continuing health education for the population. This study is of great relevance, as these barriers must be considered during the methodological planning of a neurorehabilitation trial in developing countries. Based on our findings, patients with USN should be included in the first three months to take advantage of the learning window and brain plasticity, as this would increase the rate of included patients and reduce the effect of USN spontaneous recovery. In addition, researchers should consider using a different screening method than just using a BIT-C cutoff score because other types of USN may not be included in future clinical trials if using only one assessment tool (32-34).

### Conclusion

The recruitment of stroke survivors with USN was restricted by the study design and limited financial support. Cognitive impairment, prior history of intracranial stent placement or craniectomy, and lack of transportation were the most common barriers to participation in a multicenter noninvasive brain stimulation trial of patients with USN after stroke.
